# Low sexual desire in postpartum women: associations with psychosocial factors and sexual function

**DOI:** 10.1093/sexmed/qfag066

**Published:** 2026-10-05

**Authors:** Lara Freitas Matos Costa, Heloísa Cristina Barbosa de Santana, Agostinho de Sousa Machado Júnior, Juliana Felipe da Silva Souza, Rúbia Rayanne Souto Braz, Maria das Graças Rodrigues de Araujo, Marcelo Renato Guerino

**Affiliations:** Department of Physiotherapy, Federal University of Pernambuco (UFPE), Recife, PE 50740-560, Brazil; Department of Physiotherapy, Federal University of Pernambuco (UFPE), Recife, PE 50740-560, Brazil; Department of Gynecology, Center for Medical Sciences, Federal University of Pernambuco (UFPE), Recife, PE 50740-560, Brazil; Postgraduate Program in Translational Health, Center for Medical Sciences, Federal University of Pernambuco (UFPE), Recife, PE 50740-560, Brazil; Department of Physiotherapy, Federal University of Pernambuco (UFPE), Recife, PE 50740-560, Brazil; Department of Physiotherapy, Federal University of Pernambuco (UFPE), Recife, PE 50740-560, Brazil; Department of Physiotherapy, Federal University of Pernambuco (UFPE), Recife, PE 50740-560, Brazil; Postgraduate Program in Translational Health, Center for Medical Sciences, Federal University of Pernambuco (UFPE), Recife, PE 50740-560, Brazil

**Keywords:** low sexual desire, postpartum women, female sexual dysfunction, female sexual function index, sexual health

## Abstract

**Introduction:**

Sexual dysfunction is highly prevalent during the postpartum period but remains underdiagnosed and insufficiently addressed in clinical practice. Reduced sexual desire is one of the most frequently reported sexual complaints during the postpartum period and is commonly associated with impaired female sexual function. This study aimed to determine the prevalence of low sexual desire (LSD) and identify associated factors in postpartum women receiving care in a public healthcare setting.

**Methods:**

This cross-sectional study was conducted in a tertiary university hospital in Brazil between February and December 2025. Data were collected through structured interviews using validated instruments, including the Female Sexual Function Index, Rosenberg Self-Esteem Scale, and Visual Analog Scale for pain. The primary outcome was the prevalence of LSD and its association with sociodemographic, clinical, psychosocial, sexual function, and self-esteem variables. Statistical analyses included parametric and non-parametric tests, post hoc power analysis, and Holm–Bonferroni adjustment for multiple comparisons.

**Results:**

Low sexual desire was identified in 56.25% of participants. Lower self-esteem, impaired sexual arousal, and lower overall Female Sexual Function Index scores remained significantly associated with LSD after adjustment for multiple comparisons. No significant associations were observed for age (*P* = .967), marital status (*P* = .586), mode of delivery (*P* = .940), or parity (*P* = .762).

**Discussion:**

Low sexual desire was highly prevalent among postpartum women and was primarily associated with psychosocial factors and impaired sexual function rather than sociodemographic or obstetric characteristics. Although the cross-sectional design precludes causal inference and the relatively small sample recruited from a single tertiary public healthcare center in urban Brazil may limit the generalizability of these findings beyond similar clinical settings, the results support the incorporation of routine sexual health assessment and psychosocial evaluation into postpartum care to facilitate early identification and management of women experiencing LSD.

## Introduction

Pregnancy is a unique and transformative event in a woman’s life, involving psychological, hormonal, and physical changes that prepare the maternal body for childbirth and motherhood.^1^ These changes are complex and individualized, varying among women and often associated with fears, uncertainties, and curiosity about bodily transformations.^2^ Understanding these factors is essential for improving clinical decision-making and guiding targeted interventions in postpartum care.^2^

During the pregnancy–puerperal cycle, complex physiological and hormonal changes directly influence women’s sexual health. Additionally, the pelvic floor muscles undergo biomechanical adaptations due to increased uterine weight, which may compromise muscle contractility.^3^ These mechanisms have been associated with the development of sexual dysfunctions, including dyspareunia, decreased sexual desire, reduced arousal and lubrication, and difficulty achieving orgasm. Although sexual dysfunction remains underreported, prevalence rates may reach ~50%.^4^

Epidemiological studies reinforce the magnitude of this issue. A study conducted in Australia reported that 64.3% of women experienced some form of sexual dysfunction within the first 12 months postpartum, with 81.2% reporting low sexual desire (LSD).^5^ In Brazil, ~53% of women report sexual dysfunction, with an incidence of up to 70% during pregnancy and postpartum. The most common complaints in the first 6 months postpartum include dyspareunia and reduced libido.^6^

Low sexual desire, assessed in this study using the desire domain of the Female Sexual Function Index (FSFI), represents a reduction in sexual interest. Although reduced desire is a core feature of Hypoactive Sexual Desire Dysfunction as defined by the ICD-11, the present study did not establish a clinical diagnosis because distress and diagnostic exclusion criteria were not assessed.^7^^,^^8^ Factors such as traumatic delivery, unemployment, history of abuse, low self-esteem, multiparity, dissatisfaction with a partner, and recent childbirth have been associated with reduced sexual desire in postpartum women.^9^

Despite increasing research, access to comprehensive sexual health care during the postpartum period remains limited, particularly in low- and middle-income countries, where structural and socioeconomic barriers restrict access to specialized care.^10^ Moreover, although postpartum sexual dysfunction has been extensively investigated, studies specifically examining LSD as a distinct outcome using validated multidimensional instruments remain limited. Most available studies have focused on overall sexual dysfunction rather than identifying the psychosocial and sexual function factors specifically associated with LSD during the postpartum period.^10^ Addressing this knowledge gap may improve the identification of women at greater risk of persistent postpartum LSD and support evidence-based strategies for screening and multidisciplinary management in routine postpartum care.

Therefore, this study aimed to determine the prevalence of LSD and identify associated factors in postpartum women receiving care in the Brazilian public healthcare system. Based on previous evidence that sexual dysfunction and psychosocial factors are highly prevalent during the postpartum period, we hypothesized that postpartum women with LSD would have lower self-esteem and poorer sexual function than women without the condition. The following research question was formulated: Is LSD in postpartum women associated with impairments in sexual function and lower self-esteem compared with postpartum women without LSD?

## Methods

This was a cross-sectional observational study approved by the Research Ethics Committee of the Hospital das Clínicas, Federal University of Pernambuco (protocol no. 4.840.515). For participants younger than 18 years, written informed assent was obtained in addition to written consent from a parent or legal guardian, according to institutional ethical requirements. All procedures were conducted in accordance with the Declaration of Helsinki. Written informed consent was obtained from all participants prior to enrollment. Participants were recruited between February 2025 and December 2025 at the Family Planning Outpatient Clinic of the Hospital das Clínicas, Federal University of Pernambuco (HC-UFPE). This study was conducted in accordance with the STROBE guidelines for observational studies.

Inclusion criteria were women aged 13-40 years who were within 12 months postpartum. Exclusion criteria were women who reported any type of sexual dysfunction before pregnancy, those who had difficulty understanding the questionnaires, individuals with chronic vulvar diseases, a history of oophorectomy or oophoroplasty, and those diagnosed with sexually transmitted infections. A formal sample size calculation was not performed because of the exploratory nature of the study. Potential sources of selection bias were minimized by consecutively recruiting all eligible postpartum women attending the outpatient clinic during the study period.

The sample was divided into two groups according to the cutoff score of the sexual desire domain of the FSFI (<4.28). Group A consisted of participants with LSD, while Group B included participants without LSD. Because the FSFI desire-domain score was used to classify participants into the study groups, this domain was not included in subsequent between-group comparisons of sexual function in order to avoid circular interpretation.

Participants underwent clinical evaluation, including medical history, sociodemographic assessment, and standardized measures of pain, sexual function, and self-esteem. Pain intensity was assessed using a 10-cm Visual Analog Scale (VAS). Sexual function was assessed using the Female Sexual Function Index (FSFI), a validated 19-item questionnaire developed by Rosen et al. Self-esteem was evaluated using the Rosenberg Self-Esteem Scale, originally developed by Rosenberg. All questionnaires were administered in their validated Brazilian Portuguese versions.^5^^,^^11^^,^^12^

### Statistical analysis

Statistical analyses were performed using SigmaPlot 12.0 (Systat Software Inc., San Jose, CA, USA). Data normality was assessed using the Shapiro–Wilk test, and homogeneity of variances was evaluated using the equal variance test. Variables were classified as continuous or categorical according to their nature. Continuous variables were expressed as mean ± standard deviation, and categorical variables as absolute and relative frequencies. Parametric tests (Student’s *t-*test) were applied for normally distributed variables, while non-parametric tests (Mann–Whitney test) were used for variables that did not meet normality assumptions.

Categorical variables were analyzed using the Chi-square test or Fisher’s exact test, as appropriate. Given the exploratory nature of the study and the relatively small sample size, a post hoc power analysis was conducted based on the difference observed in the FSFI total score, which represented the primary outcome of interest. Considering the final sample size (*n* = 32), an alpha level of .05, and the observed effect size (Cohen’s *d* = 0.69), the achieved statistical power was ~70%. To reduce the risk of type I error due to multiple comparisons, *P*-values for the analyses presented in Table 3 were adjusted using the Holm–Bonferroni procedure. Statistical significance was set at *P* < .05.

## Results

A total of 39 postpartum women were assessed, of whom two withdrew and five participants could not be contacted despite repeated contact attempts, resulting in a final sample of 32 participants (Figure 1). The mean age was similar between groups (Group A: 28.05 ± 6.07 years; Group B: 28.14 ± 5.50 years), with no statistically significant difference (*P* > .05). Although the inclusion criteria allowed enrollment of participants aged 13 years or older, no participants younger than 18 years were enrolled in the study.

**Figure 1:**
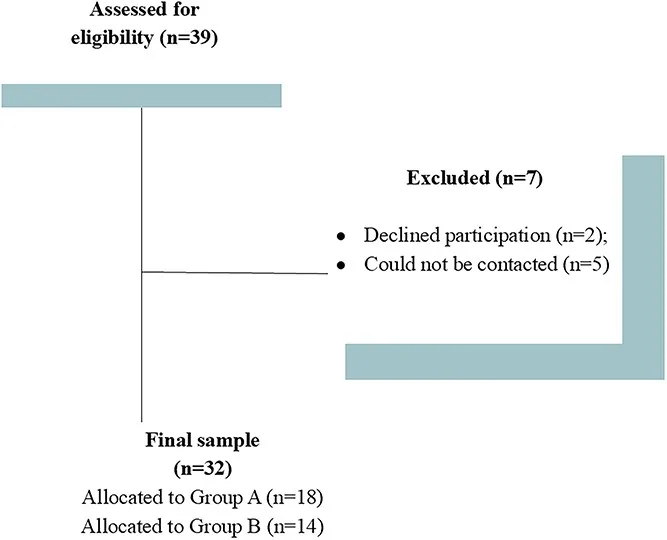
Flowchart of participant recruitment and selection. Flowchart illustrating the recruitment and selection process of postpartum women included in the study. A total of 39 participants were initially assessed for eligibility. Of these, 2 declined participation and 5 could not be contacted due to unsuccessful attempts. The final sample consisted of 32 participants, who were allocated into two groups according to the presence (Group A) or absence (Group B) of low sexual desire.

No statistically significant differences were observed between groups regarding sociodemographic characteristics, including race, education level, marital status, employment status, income, and place of residence (*P* > .05). The sociodemographic characteristics of the participants are presented in Table 1.

**Table 1:** Sociodemographic characteristics of postpartum women according to low sexual desire status (*n* = 32).

Variables	Group A (*n* = 18)	Group B (*n* = 14)	*P*-value
**Age (years)**	28.05 ± 6.07	28.14 ± 5.50	.967
**Race**			
Mixed-race	13 (72.2%)	9 (64.3%)	.520
White	3 (16.7%)	1 (7.1%)	
Black	1 (5.6%)	3 (21.4%)	
Asian	1 (5.6%)	1 (7.1%)	
**Marital Status**			
Common-law marriage	8 (44.4%)	4 (28.6%)	.586
Single	6 (33.3%)	7 (50%)	
Married	4 (22.2%)	3 (21.4%)	
**Educational level**			
Incomplete elementary education	2 (11.1%)	2 (14.3%)	.721
Completed basic education	2 (11.1%)	3 (21.4%)	
Incomplete secondary education	3 (16.7%)	0 (0%)	
High school diploma	10 (55.6%)	7 (50%)	
Technical school	1 (5.6%)	0 (0%)	
Incomplete higher education	0 (0%)	1 (7.1%)	
Complete higher education	0 (0%)	1 (7.1%)	
**Family income**			
No income	5 (27.8%)	3 (21.4%)	.312
Up to 1 minimum wage	13 (72.2%)	8 (57.1%)	
Between 2 and 5 minimum wages	0 (0%)	3 (21.4%)	
**Occupation**			
Unemployed	10 (55.6%)	7 (50%)	.186
Employed	5 (27.8%)	3 (21.4%)	
Self-employed	1 (5.6%)	4 (28.6%)	
Informal work	2 (11.1%)	0 (0%)	
**Residence**			.142
Metropolitan region	17 (94.4%)	11 (78.6%)	
Rural area	0 (0%)	2 (14.3%)	
Forest zone	1 (5.6%)	0 (0%)	
Semi-arid region	0 (0%)	1 (7.1%)	
**Sedentary lifestyle**			.418
Yes	5 (27.8%)	6 (42.9%)	
No	13 (72.2%)	7 (50%)	
Contraindication to exercise	0 (0%)	1 (7.1%)	

Data are presented as mean ± standard deviation (SD) for continuous variables and as absolute and relative frequencies (*n*, %) for categorical variables. Group A: women with low sexual desire; Group B: women without low sexual desire. Comparisons between groups were performed using Student’s *t-*test or Mann–Whitney test for continuous variables and Chi-square or Fisher’s exact test for categorical variables. Statistical significance was set at *P* < .05. The symbol (–) indicates that statistical comparison was not performed because of sparse frequencies or zero counts in one or more categories.

These results indicate that sociodemographic variables were not associated with LSD in this sample.

Obstetric and postpartum variables are summarized in Table 2. No statistically significant associations were observed between LSD and obstetric or postpartum variables, including number of pregnancies, parity, postpartum duration, prenatal care characteristics, mode of delivery, perineal trauma, and pregnancy-related complications.

**Table 2:** Obstetric and postpartum characteristics of participants according to low sexual desire status.

Variables	Group A (*n* = 18)	Group B (*n* = 14)	*P*-value
Number of pregnancies	2.6 ± 1.60	2.85 ± 1.91	.762
Number of children	2.16 ± 1.19	2.42 ± 1.74	.921
Postpartum period	4.6 ± 2.67	5.42 ± 3.69	.741
**Prenatal care**			
Yes	18 (100%)	14(100%)	–
No	0 (0%)	0(0%)	
**Location of prenatal care**			
Public service	18 (100%)	14 (100%)	–
Private service	0 (0%)	0 (0%)	
**Method of childbirth**			
Vaginal	9 (50%)	6 (42.9%)	.940
Cesarean section	9 (50%)	8 (57.1%)	
**Perineal and obstetric history**			–
Spontaneous laceration	0 (0%)	1 (7.1%)	
Episiotomy	0 (0%)	2 (14.3%)	
Abortions	0 (0%)	2 (14.3%)	
**Problems during childbirth**	7 (50%)	4 (28.6%)	.712

Data are presented as mean ± standard deviation (SD) or as absolute and relative frequencies (*n*, %). Group A: women with low sexual desire; Group B: women without low sexual desire. Variables include pregnancy-related, delivery-related, and postpartum characteristics. Comparisons between groups were performed using Student’s *t-*test or Mann–Whitney test for continuous variables and Chi-square or Fisher’s exact test for categorical variables. Statistical significance was set at *P* < .05. The symbol (–) indicates that statistical comparison was not performed because of sparse data.

Sexual function outcomes assessed by the FSFI are presented in Table 3. Women with LSD showed significantly lower scores in the FSFI domains of arousal, lubrication, orgasm, and satisfaction in the unadjusted analyses (*P* < .05). To account for multiple comparisons across FSFI domains, *P*-values were adjusted using the Holm–Bonferroni procedure. After adjustment, only the FSFI total score (adjusted *P* = .012) and the arousal domain (adjusted *P* = .015) remained significantly different between groups. The differences observed in the lubrication, orgasm, and satisfaction domains did not remain statistically significant after correction for multiple testing. These findings suggest that, after controlling for multiple comparisons, LSD remained significantly associated with impaired sexual arousal and poorer overall sexual function, whereas differences in lubrication, orgasm, and satisfaction were no longer statistically significant.

**Table 3:** Comparison of pain, self-esteem, and sexual function between groups with and without low sexual desire.

Variables	Group A (*n* = 18)	Group B (*n* = 14)	*P*-value	Holm–Bonferroni adjusted *P*-value
**Visual Analog Scale (VAS)**	1.6 ± 2.1	2.78 ± 3.5	.650	
**Current pain**			.964	
No	9 (50%)	8 (57.1%)		
Yes	9 (50%)	6 (42.9%)		
**Self-esteem scale**	28.27 ± 3.7	32.3 ± 4.5	.009	
**FSFI**				
Desire	2.73 ± 0.94	5.1 ± 1.57	–	–
Arousal	2.31 ± 1.86	4.36 ± 2.06	.003^a^	.015^a^
Lubrication	3.14 ± 2.13	4.23 ± 2.36	.030^a^	.092
Orgasm	2.44 ± 2.17	4.16 ± 2.38	.023^a^	.092
Satisfaction	2.77 ± 2.33	4.37 ± 2.43	.033	.092
Pain	3 ± 2.28	3.54 ± 2.24	.500	.500
**Average total score**	**16.17 ± 11.16**	**24.1 ± 11.85**	.002^a^	.012^a^

^a^Data are presented as mean ± standard deviation (SD) or absolute and relative frequencies (*n*, %). Group A: women with low sexual desire; Group B: women without low sexual desire. VAS: Visual Analog Scale; FSFI: Female Sexual Function Index. Comparisons between groups were performed using Student’s *t-*test or Mann–Whitney test for continuous variables and Chi-square or Fisher’s exact test for categorical variables. *P* < .05 indicates statistical significance in the unadjusted analyses. The desire-domain score was used to define the study groups; therefore, no statistical comparison was performed for this variable. Holm–Bonferroni correction was applied to FSFI domain comparisons; after adjustment, only the arousal domain and the FSFI total score remained statistically significant (See “Results”). The symbol (–) indicates that statistical comparison was not performed because of sparse data.

No statistically significant differences were observed between groups in the pain domain (*P* = .500), nor in VAS scores. Self-esteem scores were significantly lower in women with LSD compared to those without the condition (*P* = .009), indicating a strong association between reduced self-esteem and LSD.

## Discussion

The present study demonstrated a high prevalence of LSD (56.25%) among postpartum women, highlighting the clinical relevance of this condition in the postpartum period. In addition, LSD was significantly associated with reduced self-esteem and impairment in overall sexual function, suggesting a multifactorial interaction between psychological and functional components underlying sexual dysfunction.^13^ Previous studies have shown that pregnancy and childbirth may negatively affect female sexual function among women who do not seek professional assistance, and among those who do seek help, only about half have access to appropriate treatment.^14^

The prevalence observed in this study is consistent with international findings, although considerable variability exists across populations. Such differences may be influenced by sociocultural-, behavioral-, and healthcare-related factors, particularly in low- and middle-income settings. A study conducted with Iranian women reported a prevalence of 79%.^15^ In Tunisia, prevalence rates of ~33.4% have been reported.^16^ These variations across different populations may be explained by cultural and regional differences, particularly in societies with strong male dominance and practices such as arranged marriages, polygamy, and female genital mutilation, which are associated with higher rates of sexual dysfunction in women.^9^

The present findings indicate that sociodemographic variables were not significantly associated with LSD. This finding contrasts with some previous studies and suggests that sexual dysfunction in the postpartum period may be more strongly influenced by psychosocial and physiological factors rather than traditional demographic variables. However, a Brazilian study reported that prevalence increased with age and lower educational levels.^17^ To date, there is no consensus regarding the effects of these factors on sexual function, and more recent studies have reported inconsistent or unclear findings.^5^^,^^9^ It is known that sociodemographic variables influence access to sexual health education, including the search for guidance and potential care.^18^ Nevertheless, these variables may indirectly affect sexual health through their impact on access to information, healthcare services, and social support.

Similarly, no association was observed between obstetric variables—including parity, mode of delivery, perineal trauma, and postpartum duration—and LSD. These findings reinforce the hypothesis that biological and obstetric factors alone may not fully explain sexual dysfunction in the postpartum period, supporting a more comprehensive biopsychosocial model.^9^ Another important factor is perineal trauma; compared with women with an intact perineum, those with second- and third-degree perineal injuries or episiotomy are more likely to develop sexual dysfunctions such as dyspareunia and reduced sexual interest.^19^ These discrepancies may be explained by methodological differences, the use of different assessment instruments, as well as the small sample size in our study. Additionally, obstetric history variables, including previous abortions, were considered due to their potential influence on reproductive and sexual health outcomes. Although no significant association was observed in the present study, these factors remain clinically relevant and should be further explored in future research.

Furthermore, although no significant differences were observed between modes of delivery, both groups presented high rates of LSD. In agreement with these findings, one study demonstrated that both vaginal and cesarean deliveries are associated with reduced FSFI scores at three months postpartum. On the other hand, it has been reported that between six and twelve months postpartum, sexual function tends to return to near-baseline levels, indicating an association between postpartum duration and sexual function.^20^

One of the most relevant findings of this study was the strong association between LSD and low self-esteem. These findings highlight the central role of psychosocial factors in female sexual health during the postpartum period and suggest that emotional and self-perception components may be key targets for clinical intervention. These findings are supported by previous studies demonstrating that a negative self-image is a significant risk factor for sexual dysfunction.^9^ Body image dissatisfaction may represent an important mechanism underlying this association. During the postpartum period, women frequently experience substantial physical changes, including abdominal laxity, weight retention, breast alterations, and perineal scars, which may negatively influence body image and perceived sexual attractiveness. These changes can reduce self-confidence, impair sexual self-esteem, and contribute to diminished sexual desire even in the absence of significant obstetric complications. Therefore, body image should be considered a clinically relevant psychosocial factor when assessing postpartum women presenting with LSD.^21^ Consistent with both the aforementioned author and our results, a qualitative analysis observed a decrease in female sexual attractiveness and overall sexual function during the postpartum period. During this period, women frequently report concerns related to episiotomy scars, labial changes, and dyspareunia, which may reduce self-confidence and sexual desire.^22^ Therefore, it is essential to provide education to these women regarding the changes occurring during the pregnancy–puerperal cycle to reduce these concerns, as well as to offer guidance on sexual dysfunctions and encourage seeking professional help.

After adjustment for multiple comparisons, impairment in the arousal domain and the overall FSFI score remained significantly associated with LSD, whereas differences in lubrication, orgasm, and satisfaction were no longer statistically significant. These findings suggest that sexual arousal may represent the principal dimension associated with reduced sexual desire in postpartum women, while the differences observed in the remaining domains should be interpreted with caution because they were not robust after correction for multiple testing.^23^ Because multivariate analyses were not performed, it was not possible to determine whether reduced self-esteem and impaired sexual arousal represent independent contributors to LSD or whether their effects overlap. Future regression-based studies are needed to clarify these relationships.

Breastfeeding status was not assessed in the present study. Therefore, a physiological pathway mediated by lactation-associated hypoestrogenism, which may negatively influence sexual desire, arousal, and vaginal lubrication during the postpartum period, cannot be ruled out and may have contributed to the observed association between arousal impairment and LSD. The overlap of sexual dysfunctions is common and may be explained by the dynamic interaction between desire and arousal mechanisms. As the physical response progresses to orgasm, it results in feelings of satisfaction and resolution, generating positive feedback that enhances sexual desire and arousal. However, when a disruption occurs at any stage of this cycle, it may lead to alterations across multiple domains of sexual function.^24^

Interestingly, although pain during sexual intercourse was frequently reported, its intensity was generally mild according to VAS scores. This discrepancy suggests that subjective discomfort may not fully correspond to objective pain measures. However, the reasons why women do or do not seek healthcare support were not assessed in the present study.^20^

These findings have important clinical implications, emphasizing the need for routine assessment of sexual health in postpartum care. Integrating psychosocial evaluation and sexual counseling into clinical practice may improve early identification and management of LSD.^25^

Several limitations should be considered when interpreting the present findings. First, the relatively small sample size may have reduced the statistical power to detect significant differences between groups, particularly for obstetric and postpartum variables presented in Table 2. Although the post hoc power analysis indicated ~70% statistical power for detecting differences in the FSFI total score, the study may have been underpowered to identify smaller effects in secondary outcomes, particularly those related to obstetric and postpartum characteristics.

Therefore, the absence of statistically significant associations for these variables should be interpreted with caution, as type II error cannot be excluded. Recruitment was conducted among women receiving care in the public healthcare system, and the socioeconomic characteristics of this population may have contributed to difficulties in participant access and retention. In addition, because of the cross-sectional design, causality cannot be inferred between LSD, self-esteem, and sexual function outcomes; the observed associations should therefore be interpreted as correlational rather than causal. Other potentially relevant factors were not assessed. Breastfeeding status was not evaluated, despite its potential influence on sexual desire and sexual function through hormonal and psychosocial pathways. Likewise, postpartum depression was not systematically screened, although depressive symptoms are recognized determinants of female sexual health during the postpartum period. Hormonal status was also not assessed, preventing evaluation of endocrine influences on LSD. Furthermore, partner-related variables, including relationship quality, partner sexual function, and emotional support, were not collected and may have influenced the outcomes observed.

Participant losses and non-response may also have introduced selection bias. Although the reasons for non-participation were not formally investigated, discussing sexuality during the postpartum period may be a sensitive issue for some women. Additionally, the absence of multivariate analysis limited the ability to identify independent predictors of LSD. Furthermore, adjustment for multiple comparisons reduced the number of statistically significant associations, indicating that some findings from the unadjusted analyses should be interpreted as exploratory. The exploratory nature of the study should also be considered when interpreting these findings. Finally, the limited number of studies specifically addressing LSD in postpartum women restricted comparisons with the existing literature.

## Conclusion

A high prevalence of LSD (56.25%) was observed among postpartum women. Low sexual desire was significantly associated with reduced self-esteem, impaired sexual arousal, and lower overall sexual function scores. No significant associations were found between LSD and sociodemographic or obstetric variables. Several limitations should be acknowledged, including the small sample size and the absence of multivariate analysis.

Further studies with larger samples and multivariate analyses are needed to better identify independently associated factors of LSD. These findings emphasize the importance of incorporating sexual health assessment and psychosocial support into routine postpartum care.

## Supplementary Material

Highlights_qfag066
