# Supplementary material for: Low sexual desire in postpartum women: associations with psychosocial factors and sexual function
Source: Sex Med. 2026 Oct 5;14(6):qfag066. doi: 10.1093/sexmed/qfag066 (PMC13635457; doi:10.1093/sexmed/qfag066)
Supplement: Supplementary file 1 [file highlights_qfag066.docx]

**LOW SEXUAL DESIRE AND ASSOCIATED FACTORS IN POSTPARTUM WOMEN: A CROSS-SECTIONAL STUDY**

**Highlights**

• Low sexual desire affected more than half of postpartum women in this study.

• Low sexual desire was significantly associated with reduced self-esteem.

• After Holm–Bonferroni adjustment, only sexual arousal and overall FSFI scores remained significantly associated with low sexual desire.

• Sociodemographic and obstetric characteristics were not associated with low sexual desire.

• Routine postpartum care should include assessment of sexual health and psychosocial well-being.
